# Dietary β-Cryptoxanthin and α-Carotene Have Greater Apparent Bioavailability Than β-Carotene in Subjects from Countries with Different Dietary Patterns

**DOI:** 10.3390/nu12092639

**Published:** 2020-08-29

**Authors:** Begoña Olmedilla-Alonso, Elena Rodríguez-Rodríguez, Beatriz Beltrán-de-Miguel, Rocío Estévez-Santiago

**Affiliations:** 1 Department of Metabolism and Nutrition, Institute of Food Science, Technology and Nutrition (ICTAN-CSIC), C/José Antonio Novais, 10, 28040 Madrid, Spain; Rocio.estevez@ufv.es; 2 Department of Chemistry in Pharmaceutical Sciences, Faculty of Pharmacy, Complutense University of Madrid (UCM), 28040 Madrid, Spain; elerodri@ucm.es; 3 Department of Nutrition and Food Science, Complutense University of Madrid (UCM), 28040 Madrid, Spain; beabel@farm.ucm.es; 4 Department of Metabolism and Nutrition, Institute of Food Science, Technology and Nutrition (ICTAN-CSIC), Faculty of Experimental Science, Francisco de Vitoria University, 28223 Madrid, Spain

**Keywords:** β-cryptoxanthin, α-carotene, β-carotene, provitamin-A carotenoids, bioavailability, bioaccessibility, human health, nutritional status, dietary markers

## Abstract

β-carotene, α-carotene and β-cryptoxanthin are greater contributors to vitamin A intake than retinol in the human diet for most people around the world. Their contribution depends on several factors, including bioavailability and capacity of conversion into retinol. There is an increasing body of research showing that the use of retinol activity equivalents or retinol equivalents could lead to the underestimation of the contribution of β-cryptoxanthin and of α-carotene. The aim is to assess their apparent bioavailability by comparing concentrations in blood to their dietary intakes and identifying the major food contributors to their dietary intake. Dietary intake (3-day 24-h records) and serum concentrations (by HPLC) were calculated in normolipemic subjects with adequate retinol status (≥1.1 µmol/L) from our studies (*n* = 633) and apparent bioavailability calculated from 22 other studies (*n* = 29,700). Apparent bioavailability was calculated as the ratio of concentration in the blood to carotenoid intake. Apparent bioavailabilities for α-carotene and β-cryptoxanthin were compared to those for β-carotene. Eating comparable amounts of α-carotene, β-cryptoxanthin and β-carotene foods resulted in 55% greater α-carotene (95% CI 35, 90) and 686% higher β-cryptoxanthin (95% CI 556, 1016) concentrations than β-carotene in blood. This suggests differences in the apparent bioavailability of α-carotene and β-cryptoxanthin and even larger differences with β-cryptoxanthin, greater than that of β-carotene. Four fruits (tomato, orange, tangerine, red pepper) and two vegetables (carrot, spinach) are the main contributors to their dietary intake (>50%) in Europeans.

## 1. Introduction

Vitamin A is an essential nutrient that is obtained through diet either as retinol from animal products or as provitamin A carotenoids mainly from plant products. Provitamin A carotenoids are greater contributors to vitamin A intake than retinol in the human diet for most people worldwide [1] and are mainly supplied by vegetables and fruits. The most abundant provitamin A carotenoid is β-carotene; α-carotene is usually present in the same foods as β-carotene but in lower concentrations. β-cryptoxanthin is present in a limited number of orange-colored fruits and vegetables [2,3]. In general, as the socioeconomic level of the population increases, a larger proportion of vitamin A comes from animal sources [4]. For instance, in Spain’s most recent national survey of adults, retinol from animal sources accounted for 58% [5] and, in the Dominican Republic, which has a high prevalence of inadequate vitamin A intake, according a national survey of household income and expenditure, foods of animal origin accounted for 20% of vitamin A intake [6], although that figure reached 41% in an upper-middle class group of Dominicans [7].

The relative contribution of the provitamin A carotenoids to the vitamin A dietary intake depends not only on the amounts of fruits and vegetables consumed and on their proportion with respect to retinol intake from animal sources, but also on the bioavailability and capacity of conversion into retinol of the carotenoids consumed. The bioavailability of the three dietary carotenoids with provitamin A activity differs depending on many factors, which can be intrinsic, i.e., subject-related such as physiological state, retinol homeostasis and sex, or diet-related, i.e., chemical state of the food components, amount ingested, interactions with components of the diet, color of the fruit or vegetable [1,8,9,10]. Moreover, carotenoid bioaccessibility and, by extension, their bioavailability from foods vary widely for different carotenoids in a given food and for a given carotenoid in different foods [11]. When assessing bioavailability, both the fraction ingested and absorbed as well as the fraction cleaved in the enterocyte and other tissues by BCO1 and BCO2 to produce retinol and other apocarotenoids must be considered [10,12].

Evaluation of diet suitability and of the risk associated with excessive or inadequate vitamin A intakes is based on nutritional status assessment using biochemical markers (retinol in blood) or dietary estimates. Retinol concentration in serum/plasma is used as a biochemical biomarker of vitamin A status as it is related to liver vitamin A concentration (considered the gold standard for vitamin A status), although retinol in blood does not reflect dietary intake of this vitamin, in well-nourished subjects, as it is subject to homeostatic control. Nutritional status data based on food intake are highly useful in taking public health decisions and in the context of epidemiological studies. However, their validity is limited for several reasons mainly related to food intake assessment, and the way in which data are presented in the food composition table (FCT) [13], as well as how vitamin A activity is measured (retinol equivalents, RE or retinol activity equivalents, RAE), among others. Current assumptions regarding RAE or RE provided for the major provitamin A dietary carotenoids (β-carotene, β-cryptoxanthin, α-carotene), based on their bioavailability from foods, consider β-carotene as a major contributor to the vitamin A intake and that the other two provitamin A carotenoids have the same bioavailability and potential for conversion to retinol, which is half that of β-carotene [14]. Thus, when the vitamin A activity is measured as RE or as RAE, in the latter form, the contribution of the provitamin A carotenoids is half that assumed when RE are used [15,16,17]. More and more research shows that the use of RAE or RE could lead to underestimating the contribution of β-cryptoxanthin and of α-carotene [9,16,18]. There are intervention studies involving foods rich in β-cryptoxanthin that lead to better serum retinol responses than β-carotene supplements [9] and also bioaccessibility studies in which β-cryptoxanthin seems to be more efficiently absorbed and/or converted into retinol than the carotenes (depending on the type of food) [18,19].

Results of vitamin A nutritional status assessments are controversial insofar as they differ depending on the markers used. For instance, studies assessing dietary intake in the Spanish population concluded that vitamin A intake is inadequate as it falls below the recommended dietary allowances values [20,21,22,23]. The most recent Spanish National Dietary Survey of adults [22] found that men’s vitamin A intake was under 80% of the recommended intake, and in a recent representative sample of the Spanish population (2009 individuals, age 9–75 years), it was found that 60% did not meet the European Food Safety Authority (EFSA)-recommended intakes for vitamin A (RE) [23]. However, there are no studies reporting deficiency when assessing the retinol concentrations in blood, and in the studies where this biomarker is reported in apparently healthy subjects, concentrations far from deficiency are described. Thus, retinol concentration in blood was in the upper end of the reference range in studies involving Spanish groups (1.61 µmol/L retinol in blood vs. 699 µg RAE dietary intake, *n* = 50, 21–30 years) [24], 1.82 and 2.38 µmol/L retinol in blood, *n* = 893, 18–74 years [25]), even for those having an inadequate dietary intake, and 1.9–2.1 µmol/L retinol in blood, *n* = 64, 25–45 years [26].

In this study, we assessed the provitamin A carotenoids’ apparent bioavailability as proposed by Burri et al. [16], while also identifying the major food contributors to their dietary intake in four of our studies on human subjects. We analyzed provitamin A carotenoids serum and dietary intake from blood samples and dietary food recalls obtained from three previous studies focused on lutein and zeaxanthin markers in Spanish subjects. Moreover, we used carotenoid concentration data in blood and dietary intake obtained in a study involving healthy European volunteers from five countries [26,27]. In addition, we searched through the scientific literature published in the last ten years and selected articles with individual provitamin A carotenoid data, in blood and diet, and calculated their apparent bioavailabilities. We compared the apparent bioavailability of β-cryptoxanthin and α-carotene with that of β-carotene to evaluate the relative importance of each of these in the dietary assessment of vitamin A nutritional status in subjects with adequate retinol nutritional status.

## 2. Materials and Methods

### 2.1. Subjects and Study Designs

**Figure 1:**
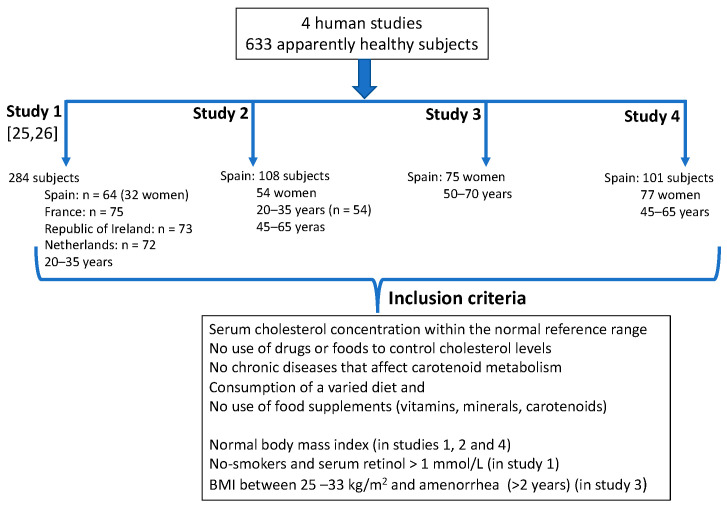
Human studies conducted by our research group.

We conducted four human studies between 1998 and 2018 involving a total of 633 apparently healthy subjects (348 Spanish subjects: 238 women, 110 men), in two age groups: 20 to 45 and 45 to 70 (Table 1). The main purpose of those studies was (Figure 1): (a) to describe dietary intake and blood concentrations of carotenoids and other antioxidant compounds in well-defined groups from five European countries (Study 1) [26,27], (b) to assess dietary and status markers of lutein and zeaxanthin in normolipemic subjects (Studies 2 and 4) [28,29] and to assess the effects of lutein and zeaxanthin and anthocyanin supplementation on lutein status markers (Study 3) [30]. Inclusion criteria for all these studies: serum cholesterol concentration within the normal reference range, no use of drugs or foods to control cholesterol levels, no chronic diseases that could affect the carotenoid metabolism, a varied diet and no use of food supplements (vitamins, minerals, carotenoids). Additionally, body mass index (BMI) within the normal reference ranges (studies 1,2,4) [26,28,29] and BMI between 25–33 kg/m^2^ and amenorrhea (>2 years) (Study 3) [30], non-smokers and serum retinol >1 mmol/L [26]. Following are the number of volunteers taking part in these studies (Figure 1). Study 1 [26,27]: 64 Spanish subjects (32 men, 32 women), and 285 from four other European countries (75 from France, 73 from the Republic of Ireland, 72 from the Netherlands and 65 from Northern Ireland), aged 25–45 years. Study 2 [28]: 108 Spanish subjects (54 men, 54 women), aged 20–35 years (*n* = 54) and 45–65 (*n* = 54). Study 3 [30]: 72 post-menopausal Spanish women, age 50–70. Study 4 [29]: 101 Spanish subjects (24 men, 77 women), aged 45–65. The studies’ approvals were obtained from each local Ethical Committee and from the Ministry of Health in Spain (Study 1) [26], from Clinical Research Ethics Committee of Hospital Universitario Puerta de Hierro-Majadahonda of Madrid, Spain (registry No. 257, dated 19 July 2010) (Study 2), from the Ethics Committee for Clinical Research of the Hospital Universitario Puerta de Hierro-Majadahonda (Madrid, Spain) (Acta No 283, dated 17 December 2012) and the Bioethics Committee of the Spanish National Research Council (CSIC) (dated 30 May 2014) (Study 3) and from the Research with Drugs of the Hospital Universitario Puerta de Hierro Majadahonda of Madrid, Spain (acta No 03.17, dated 13 February 2017) and by the Bioethic Subcommittee-Ethics Commitee (CSIC) (dated 21 February 2017) (Study 4). All subjects gave their written informed consent after receiving oral and written information concerning the studies.

The provitamin A serum concentrations and dietary intake from Study 1 were previously published [26,27]. In this study, we analyzed the provitamin A carotenoids in serum samples and evaluated their dietary intake from blood samples and food records obtained from participants in studies 2–4 (study designs published in [29,30]).

### 2.2. Dietary Carotenoid Intake Assessment

Individual dietary carotenoid intake was assessed by means of a food frequency questionnaire (FFQ) in one study (Study 1) [27] and three-day 24-h dietary recalls in the other three studies (Studies 2–4). Study 1: Semi-quantitative FFQ, containing 107 food items. Carotenoid intake was assessed for the whole diet, and the individuals were asked about their dietary intake of foods over the three previous months. A carotenoid food composition database was compiled for this study from a variety of sources [27], one of these sources being the data generated in our laboratory [31,32].

Studies 2–4: three-day food records involving 24-h recalls, one of which coincided with a Sunday or holiday, carried out over a period of 7 to 10 days. The first was completed during a personal encounter with a trained interviewer, coinciding with blood sample collection. Dietary records were assessed using a specific software application for carotenoids [33] that includes a carotenoid database, developed by our group [2], which contains information on the major dietary carotenoids present in foods commonly consumed in Spain, along with data generated by high-performance liquid chromatography (HPLC) [31,32]. Carotenoid intake was assessed for the entire diet.

### 2.3. Carotenoid Analysis in Serum

Fasting venous blood samples were taken and serum was separated by centrifugation. In Study 1, carotenoid extraction was carried out on serum samples using a previously published method [34], and in the other three studies using another published method [35], with slight modifications.

β-Carotene, α-carotene and β-cryptoxanthin were determined by HPLC using a system consisting of a model 600 pump, a Rheodyne injector and a 2998 photodiode array detector (Waters, Mildford, MA, USA). The chromatographic system included a Spheri-5-ODS column (Applied Biosystems, San Jose, CA, USA) used with a gradient elution of acetonitrile: methanol (85:15) for 5 min to acetonitrile:methylene chloride:methanol (70:20:10) for 20 min, flow rate 1.8 mL/min (in Studies 1 and 2) and a C30 YMC column (Waters, Wilmington, MA, USA) with a mobile phase of methanol with 0.1% trimethylamine: methyl-tert-butyl-ether (MTBE) in a linear gradient from 95:5 to 70:30 in 30 min, to 50:50 in 20 min; this proportion was maintained for 35 min, flow rate mL/min (in Studies 3 and 4).

During sample analysis in Study 1 [26], the accuracy and precision of the analytical method employed for carotenoids and retinol was contrasted periodically through our participation in the Quality Assurance Programme conducted by the National Institute of Standards and Technology (NIST; Gaithersburg, MD, USA), and the performance of the results was within 1 ± 2 standard deviations from NIST-assigned values (rated by NIST as exceptional or acceptable values). In Studies 2 and 3, NIST 968e reference material was analyzed, and results for β-carotene and β-cryptoxanthin were within 1 standard deviation from NIST assigned values.

### 2.4. Literature Review

We searched the PubMed database between January 2010 and May 2020 to identify studies that reported individual data of provitamin A carotenoid data in dietary intake and in blood concentrations. Literature searches were limited to human studies, observational and intervention baseline studies. The following keywords were used: cryptoxanthin, carotene, diet and serum, plasma or blood. We found 109 articles, only 21 of which reported individual carotenoid data on dietary intake and blood (serum or plasma). From these studies, we extracted only the data corresponding to apparently healthy subjects (see other health conditions in Table 2).

### 2.5. Mathematical Analysis and Statistics

Estimated dietary carotenoid intake and blood concentrations were converted into µmol/day and µmol/L, respectively. The following conversion factors were used to transform µg/dL into µmol/L: ×0.01863 (for α-carotene and β-carotene) and ×0.01810 (for β-cryptoxanthin) and to transform µg/day into µmol/day divided by 536.87 (for α-carotene and β-carotene) and by 552.85 for β-cryptoxanthin.

The blood level of each carotenoid was divided by its dietary intake to calculate the apparent bioavailability, a measurement that provides an idea of the amount of the dietary carotenoid that is absorbed or converted into retinol. This is estimated by dividing the carotenoid concentrations in the blood by dietary intake of that carotenoid (bioavailable carotenoid (bio) in the blood, e.g., α-carotene-blood/α-carotene-diet = α-carotene^bio^) [16]. From among the carotenoids, β-carotene is the major source of vitamin A and is therefore used as the standard for comparison (α-carotene^bio^/β-carotene^bio^ and β-cryptoxanthin^bio^/β-carotene^bio^).

Descriptive data are shown as means or medians. Ratios between carotenoid concentrations in serum and diet were estimated using the Student’s *t* and Wilcoxon test. Statistical significance was set at *p* < 0.05, and analyses were performed with IBM SPSS Statistics 25.0 (Armonk, NY, USA; IBM Corp).

## 3. Results

Dietary intakes (median values) and serum concentrations (mean values) of provitamin A carotenoids in our studies in subjects from Spain and four other European countries (samples analyzed in our lab) are shown in Table 1. Dietary intake was assessed by three 24-h food recalls [28,29,30] or a semiquantitative FFQ [27] and the same [28,29,30] or similar [27] carotenoid food composition databases were used in all four of these studies. Blood samples were obtained at the same time as the dietary assessment. In these healthy subjects (age range 20–70), intake medians ranged from 0.3 to 2.3, 2.3 to 10.9 and 0.3 to 2.5 µmol/day for α-carotene, β-carotene and β-cryptoxanthin, respectively. Median dietary intakes of β-carotene and α-carotene were correlated with one another (*r* = 0.899, *p* = 0.002). The dietary intake of β-carotene was greater than that of β-cryptoxanthin (*p* = 0.002) which, in the individual studies, was greater or similar to that of α-carotene but, considering the entire sample, did not reach the statistical significance. Serum concentration means in these normolipemic subjects ranged from 0.08 to 0.17, 0.37 to 0.71 and 0.23 to 0.52 µmol/L for α-carotene, β-carotene and β-cryptoxanthin, respectively. α-carotene and β-carotene serum values also correlated with one another (*r*= 0.857, *p*= 0.007). As in the case of dietary intake, β-carotene was present in serum in greater concentrations than in the other two (β-carotene in higher concentrations than β-cryptoxanthin, *p* = 0.005 and β-cryptoxanthin in higher concentrations than α-carotene (*p* = 0.000)). No significant correlations were found between the concentrations of these carotenoids in diet and in serum.

No age-related difference was found in the dietary intake of α-carotene and β-cryptoxanthin in our studies. However, β-carotene intake was significantly higher in younger subjects (age 20–45) compared to their older counterparts (age 45–70) (*p* = 0.053). No differences were found in the serum concentration of any of the provitamin A carotenoids.

Table 2 shows the dietary intake of provitamin A carotenoids compiled from the literature [16,36,37,38,39,40,41,42,43,44,45,46,47,48,49,50,51,52,53,54,55]. Data are listed by geographical origin and by the subpopulations studied in the original articles when reported by age or sex. In most of these studies, dietary intake assessment and blood extraction were obtained from the same volunteers, although some studies were part of large surveys (e.g., The National Health and Nutrition Examination Survey (NHANES) [44]) and intake and blood carotenoid data did not necessarily come from the same volunteers, which explains why a higher number of dietary assessments than blood samples were reported in these studies. α-carotene and β-carotene dietary intake and serum concentrations were within the range of those described for Spanish healthy subjects, but this was not the case for β-cryptoxanthin concentrations in diet and blood. Although β-cryptoxanthin concentrations were lower, the only significant difference was in blood concentrations (*p* = 0.013).

The dietary assessment method and the FCT used in the studies are shown in Table 1 and Table 2. In our studies, the 24-h food recall (triplicate) and the FFQ were used, and the FCT was practically the same in the four studies. In the studies compiled from the literature, a wider range of dietary assessment methods were used, although the most frequent were the FFQ and the 24-h recall/records (obtained during a different number of days, from 1 to 30 days). Moreover, different FCTs were used, and in some of the studies, this is not mentioned. Where dietary intake was assessed using more than one method for the same study’s participants (in 5 out of 22 studies), carotenoid intake assessed by 24-h recall was selected to obtain the mean values appearing in Table 2 because FFQ tends to overestimate the dietary intake of carotenoids [16,35,43].

Table 1 and Table 2 show the estimated amount of each provitamin A carotenoid absorbed and circulating in the bloodstream, i.e., the apparently bioavailable carotenoids. The median values of apparently bioavailable β-cryptoxanthin in our studies are similar to those calculated using data obtained from the literature (0.67 and 0.71, respectively). However, those of α-carotene and β-carotene are higher in our studies, both for Spanish subjects (*n* = 348) and the whole sample, which includes subjects from other European countries (*n* = 633) (α-carotene^bio^ 0.32 and β-carotene^bio^ 0.17) compared with the median values from the literature which include people from nine countries and four continents (*n* = approximately 30,000) (α-carotene^bio^ and β-carotene^bio^ 0.10). However, when comparing our data from Spanish subjects with those from other nationalities, a significant difference was found only for α-carotene^bio^ (*p* = 0.007).

Combining all data shown in Table 1 and Table 2, the apparent bioavailability of each of the three provitamin A carotenoids is lower than 1, a value that would mean a complete bioavailability, the median for β-cryptoxanthin (0.635) being quite a bit higher than for α-carotene (0.110) (*p* = 0.000) and for β-carotene (0.095) (*p* = 0.000). The apparent bioavailabilities of α-carotene and β-carotene were also statistically different (*p* = 0.001). In the homogeneous sample of Spanish subjects, bioavailability followed the same pattern, β-cryptoxanthin > α-carotene > β-carotene (medians: 0.69, 0.32 and 0.14, respectively). In this subsample, β-cryptoxanthin bioavailability exhibited statistical differences with respect to β-carotene (*p* = 0.043) but not with respect to α-carotene (*p* = 0.08). There were also differences between β-carotene and α-carotene (*p* = 0.041).

The α-carotene^bio^/β-carotene^bio^ and β-cryptoxanthin^bio^/β-carotene^bio^ ratios in the 34 studies where such data could be calculated are shown in Table 1 and Table 2. Although ratios varied widely between studies, α-carotene^bio^/β-carotene^bio^ was equal to or greater than 1 with just four exceptions (less than 1), the range being 0.5–3.75 and the mean 1.62 (CI 95% 1.35, 1.90). The median ratio in the studies performed in our lab was 1.63 (8 groups of subjects) and 1.35 in the compiled studies (28 groups of subjects). A higher ratio was obtained for β-cryptoxanthin^bio^/β-carotene^bio^, which was always greater than one, the range being 1.85–29.5, the mean 7.86 (CI 95% 5.56, 10.16) and the median 4.72 in the studies carried out in our lab and 8.38 in the compiled research. Hence, the intake of comparable amounts of α-carotene and β-carotene would result in a 55% higher concentration of α-carotene in the blood, and the intake of comparable amounts of β-cryptoxanthin and β-carotene would result in a 686% higher concentrations of β-cryptoxanthin in the blood.

These ratios of apparent bioavailability ratios indicated clearly higher bioavailability of β-cryptoxanthin and α-carotene in comparison to β-carotene. However, there are doubts regarding the comparability of these results mainly due to the high variability of the dietary assessment method employed and to the variability of the FCT (not always supplied). We tried to minimize the variability arising from the dietary assessment method, and results from the 24-h recalls were selected for the statistical analysis.

The dietary carotenoid intake assessment must consider both the carotenoid content of the food consumed and which foods are consumed, given that the food matrix leads to different carotenoid bioaccessibilities [11]. We therefore assessed the contribution of the foods consumed by the participants in our studies to the intake of each provitamin A carotenoid. Table 3 shows the major food contributors to dietary provitamin A intake and the percentage of contribution to the dietary intake of the individuals included in our studies, mainly Spanish but also groups from four other European countries. In general terms, six fruits and vegetables account for more than 50% of the dietary intake of these three carotenoids: α-carotene (carrot), β-carotene (carrot, spinach, tomato), β-cryptoxanthin (orange y orange juice, tangerine, red pepper).

## 4. Discussion

In this study, we approached the bioavailability assessment of the individual provitamin A carotenoids by means of their dietary intake and concentrations in blood in apparently healthy and normolipemic adults (age 20–75) and identified their major food contributors in their dietary intake. Dietary intake showed a wide range of concentrations, mainly for β-carotene, as described in other studies [8,27,56], and we therefore reported the median intake values to gain a more realistic idea of the intake. However, in the literature, mean rather than median values are typically reported as can be observed from the data compiled in Table 2, sometimes making it difficult to draw comparisons. The proportions of each carotenoid in the dietary intake in these Spanish groups (*n* = 348, age range 20–75) are 75.5% for β-carotene, 15.3% for β-cryptoxanthin and 9.2% for α-carotene. These values are practically identical to the ones published in the most recent Spanish National Dietary Intake Survey (*n* = 3000, age range 18–64) on β-carotene (71.9%), β-cryptoxanthin (15.3%) and α-carotene (12.8%) [5]. Similar proportions were obtained from data compiled from other studies in nine countries around the world, with β-carotene accounting for 79.2% of the provitamin A carotenoid intake and β-cryptoxanthin and α-carotene 15.3% and 5.5%, respectively. These similarities are despite important variables that could have had an impact on the dietary carotenoid intake data from the studies compiled from the literature, as different methods of assessment methods and FCTs [13] were used. In contrast, in our data from Spanish groups, the results of the same dietary assessment method and the carotenoid composition tables in all three studies are quite similar, as carotenoid composition data from Spain were included in the carotenoid food composition data of the European study [27].

These carotenoids profile proportions follow the same pattern in dietary intake as in blood (β-carotene > β-cryptoxanthin > α-carotene). There was a correlation between α-carotene and β-carotene in dietary intake and blood concentrations as in other studies [56], as can be expected because both are supplied by the major dietary food contributors (Table 3) and are often found in the same foods [3]. In contrast, β-cryptoxanthin is mainly supplied by red-orange fruits and juices [3,5]. In the analysis of carotenoids in blood in the compiled studies, serum or plasma were reported as matrices for analysis, an irrelevant aspect as an acceptable degree of agreement was described in results obtained using these two matrices [57]. In Spanish subjects, the β-carotene accounts for 51% of the total provitamin A carotenoids, β-cryptoxanthin for 37% and α-carotene for 12%. A similar pattern was found in the mean values of the compiled studies, 60% β-carotene, 25% β-cryptoxanthin and 15% α-carotene. Although the proportions of β-cryptoxanthin and α-carotene in blood are similar in some studies [39,45,55], in others, β-cryptoxanthin concentration is higher than that of α-carotene in blood but not in the dietary intake [56], or the percentage of blood β-cryptoxanthin is half that of α-carotene [48] and β-cryptoxanthin is slightly higher than β-carotene [37]. These differences are clearly seasonal in some population groups [8,56] but could also be due to factors related to the subjects included in the studies since, although in general, no characteristics were reported that could affect carotenoid metabolism, there could be exceptions. For instance, carotenoid concentrations in blood may be not comparable in normo- or hypercholesterolemic subjects [26,28] or in subjects with deficient or adequate vitamin A nutritional status, as their conversion into retinol depends on retinol concentration in the bloodstream, among other factors [14,17]. These two sources of variability could be considered as negligible in the Spanish study results as all participants were normolipemic and had an adequate retinol status as assessed by retinol serum concentrations (greater than 1.1 µmol/L) [1]. Blood retinol concentrations were ≥1.9 µmol/L [26], >1.4 µmol/L (unpublished data, from subjects in Study 3 [30]) and >1, 1 µmol/L (unpublished data, from studies in Studies 2 and 4 [28,29]). Other potential modifiers of the carotenoid status are weight (subjects were normo- and overweighted in Burrows et al. [55], in some of the studies from Burri et al. [16] and normoweight [26,27,28,29]) and normoweighted or preobesity [30] in Spanish studies, and diabetes [50] as higher concentrations of provitamin A carotenoids in insulin-dependent diabetics have been reported [34,35]. The consumption of food supplements is on the rise, and these foods used to include β-carotene in their formula and thus would modify its concentration in blood. The consumption of food supplements/vitamins was an exclusion criterion in the studies involving Spanish subjects [26,27,28,29] but not in those from Burri et al. [16] where subjects could have consumed a daily multivitamin supplement containing up to 1–2 mg of β-carotene.

The α-carotene^bio^/β-carotene^bio^ ratios (medians of 1.63 and 1.35, from our lab and from literature, respectively) and β-cryptoxanthin^bio^/β-carotene^bio^ (medians of 4.72 and 8.38, from our lab and from literature, respectively) were obtained from a larger group of subjects than those analyzed in the studies done by Burri et al. [16] (*n* = 633 vs. 229) and in more than a half of the compiled studies from the literature (since year 2010), and similar results were obtained as eating similar amounts of α-carotene and β-carotene from different dietary patterns around the world (Table 2) resulted in about 62% (1.62, CI95% 1.35, 1.90) higher α-carotene blood concentrations (vs. 53% [16]) and 686% (7.86, CI95% 5.56, 10.16) higher β-cryptoxanthin blood concentrations (vs. 725% [16]). This greater apparent bioavailability for these two provitamin A carotenoids was found despite a number of methodological factors that could account for differences in the data obtained, mainly from the dietary assessment as carotenoids concentrations in blood were, in all studies, analyzed by HPLC, thus making them comparable.

Dietary carotenoid intake estimation involves two important aspects which make it hard to draw a comparison between studies and lead to a poor reliability in terms of intake. These are the dietary carotenoid assessment method and the FCTs used. From among the carotenoid intake assessment methods, the FFQ and the 24-h food records/recalls were the one most frequently used, but even within each method, there were differences such as a variable number of items included and the different number of recorded days. These and other differences, such as the validity of the questionnaires, the food coding/identification and the conversion of the dietary intake into grams, have an impact on the comparability of the results [13]. Thus, as dietary carotenoid intake is usually overestimated when using FFQ compared with the three-day food record [16,35,43], except β-cryptoxanthin [35], for the statistical analysis, we selected the results obtained from food records when more than one dietary assessment method was applied to the same subjects. As most of the compiled studies provide no information with regard to carotenoid FCTs/databases, it is impossible to know whether carotenoid content data were obtained by HPLC or spectrophotometrically or if the xanthophyll content refers to free or total forms. These are some of the relevant topics affecting data reliability that need to be considered when selecting a carotenoid FCT [3,58].

Thus, in many studies, the greater apparent bioavailability of α-carotene over β-carotene could be the result of underestimating intake because data are lacking on foods found in smaller concentrations than β-carotene and in fewer foods [3,16]. However, the α-carotene^bio^/β-carotene^bio^ ratio for Spanish subjects (*n* = 348, ratio 2.1) was not affected by this potential underestimation or was affected only slightly, as the same database of carotenoid content in foods was used in all of those studies and contains data on the six major dietary carotenoids in frequently consumed Spanish foods [2,31,32]. However, physiological mechanisms cannot be ruled out as interactions between β-carotene and α-carotene have been described, suggesting that these carotenes could follow similar pathways to cellular uptake and/or incorporation into chylomicrons [59].

Although β-carotene is considered the major contributor to the vitamin A intake, and half of this capacity is attributable to α-carotene and β-cryptoxanthin [14], in different types of studies, β-cryptoxanthin appears to be more efficiently absorbed and converted into retinol than the carotenes [9,60], probably because xanthophyll esters, from fruit and vegetables, are more bioaccessible than carotenes [61] or free forms [62]. Moreover, dietary intake of β-cryptoxanthin has probably also been underestimated in most studies as it is mainly present as ester forms, and few FCT have included its concentrations in foods. Indeed, the presence of carotenoid esters has often been overlooked [3,62] despite their higher stability during food processing and the fact that these ester forms facilitate solubilization and extraction (bioaccessibility) during digestion [63,64]. Methodological issues could account for a portion of the high β-cryptoxanthin^bio^/β-carotene^bio^ ratio obtained from the data compiled from the literature (median: 8.4) but are less likely to totally account for that found among Spanish subjects (median: 3.4), as in our studies, this underestimation is expected to be minimal because in the dietary intake assessment, we used a common carotenoid food content database of Spanish foods, and xanthophylls esters were taken into account in the analysis of the total content. Other factors that should be taken into account are the seasonal variation in the serum concentrations of some carotenoids (i.e., β-cryptoxanthin) [3,62] and their potentially different half-life in serum.

Lastly, as the carotenoid bioavailability to reach the bloodstream depends in part on the type of carotenoid and the food matrix [11], we identified the food contributors and the percentage of their contribution to the dietary intake of each of these carotenoids. In the Spanish subjects, the major food contributors were two vegetables (carrots, spinach) and four fruits (tomato, orange, tangerine, red pepper), five out of the six of which are red-orange-colored fruit and vegetables. This color group of food plants was the major contributor to dietary provitamin A in the last National Spanish Survey of dietary intake [5] that included mainly fruits [65]. Carotenoids in fruits are found mainly in chromoplasts and are more efficiently released by digestion than carotenoids in green vegetables, which are mainly located in chloroplasts [62].

β-cryptoxanthin is present in our diet in both free form and esterified with fatty acids, and although there is very little information on those ester forms, dietary xanthophylls are ingested in a much higher percentage as ester forms (two or three times higher than the free form [11,64,66]), for which higher bioavailability and higher conversion to retinol have been described [62]. In our studies, β-cryptoxanthin is supplied mainly by red-orange foods (orange, tangerine and red peppers), and its bioaccessibility/bioavailability have been the topic of several studies, with quite different results mainly due to the use of methods that vary widely in their use of the factors involved in the simulation of the digestion process and in the way in which results are expressed [18]. Thus, bioaccessibility of 6–10% [67] and 42% [11] is reported for orange and 53% for orange juice [68]. In pepper, it ranges from 2% to 17% [69,70], 44.5% [71], 98% [11] and in mandarine 20% [67].

Regarding α-carotene, although there are fewer data regarding carrots, its major contributor, bioaccessibilities of 25%, 38% and 72% have been reported [11,72,73], and for banana, the percentage of bioaccessibity is in the range of 14–41% [74]. Red-orange fruits and vegetables contain α-carotene in similar percentages as β-cryptoxanthin in the Spanish diet [5]. A greater variety of foods contribute to the dietary intake of β-carotene, and they are mainly red-orange fruits and vegetables, as previously reported in the Spanish diet, in which they supply 64% [5]. Different bioaccessibility percentages have been reported for β-carotene contained in carrot (12% [72], 37% [73], 77% [11]), in tomato (62% [73]), red pepper (0.4–13% [69,71], 30% [72] and 71% [11]) and spinach (12% [72], 25% [11]).

Estimation of the bioavailability of dietary provitamin A carotenoids based on dietary intake data and concentrations in blood has several limitations, the most relevant probably being the disparity in the dietary intake assessment methods and FCT (referred to above), and the fact that it is impossible to estimate their conversion into retinol in the human intestine and other tissues. The extent of this conversion varies widely among individuals, depending on diverse host related factors, such as the vitamin A nutritional status, the nucleotide polymorphisms (SNPs) within the b-carotene 15,15′-monoxygenase and specific binding proteins involved in their metabolism, presence of disease, seasonal variation in the food intake, sex, age and body mass index [12,14,75,76,77]. Strengths of this study include the homogeneous characteristics of the subjects in our four studies with respect to (normal) cholesterolemia and to (adequate) retinol nutritional status. However, we found no information on these two variables in the articles from the literature that we used to calculate apparent bioavailability, either in general on in the FCT. Nonetheless, apparent bioavailabilities of the three provitamin A carotenoids showed similar results obtained from studies in very different groups of people, with different dietary intake patterns, age, sex, race, etc. Another limitation related to the dietary intake of these carotenoids is that none of the studies assessed the intake of the commercial foods fortified/enriched with vitamin A (mainly added as β-carotene), meaning that dietary intake may be underestimated. Lastly, it should be recalled that none of the studies from which we calculated the apparent bioavailability were designed for this purpose.

## 5. Conclusions

Currently, the vitamin A intake is expressed as RE or RAE, and it is assumed that α-carotene and β-cryptoxanthin contribute in the same proportion to this intake, and these account for half the contribution of β-carotene [14]. This study looks into the contribution made by α-carotene and β-cryptoxanthin to the dietary intake of vitamin A considering that the apparent bioavailability of α-carotene and, to a greater extent, that of β-cryptoxanthin are greater than that of β-carotene and differ from one another in subjects with cholesterolemia within the normal range and an adequate level of blood retinol. Use of RAE and RE appears to lead to underestimation of the contribution of two of the three major dietary provitamin A carotenoids in subjects around the world. More intervention studies in subjects with different vitamin A nutritional status, mainly involving foods rich in β-cryptoxanthin, are needed to verify whether this is really more efficiently absorbed and/or converted into retinol than β-carotene. Preliminary evaluation of their bioaccessibility from different food matrices could be helpful in the selection of foods to be used in the intervention studies.

The following four fruits and two vegetables contribute more than 50% of the dietary intake of the three provitamin A carotenoids in European subjects: tomato, orange and orange juice, tangerine and red pepper, on the one hand, and carrot and spinach on the other. Therefore, any variation or culinary/manufacturing process applied to these foods resulting in an increase in their bioavailability would have a great impact in the vitamin A nutritional status of populations with presumably different dietary patterns.

## Figures and Tables

**Table 1:** Dietary intakes (median) and serum concentrations (mean) of α-carotene (α-car.), β-carotene (β-car.) and β-cryptoxanthin (β-cx) in Spanish and other European normolipemic subjects (20–70 years).

		Mean Concentration in Blood (µmol/L)			Median Dietary Intake (µmol/day)			Apparent Bioavailability			Ratio of Apparent Bioavailability		
Date, [Ref.]	Country, n Subjects, (Sex), Age	α-Car.	β-Car.	β-Cx.	α-Car.	β-Car.	β -Cx	α-Car.^bio^	β-Car.^bio^	β-cx.^bio^	α-Car.^bio^/β-Car.^bio^	β-Cx^bio^/β-Car.^bio^	Dietary Intake Assessment Method/FCT
2020 ^1^ [29]	Spain												3 × 24-h food recalls Own database [2,32]
	101, (77 w, 24 m) 45–65 years	0.17	0.66	0.52	0.53	3.38	0.87	0.32	0.2	0.6	1.6	3	
2018 ^1^ [30]	Spain												3 × 24-h food recalls Own database [2,32]
	75 (w) 50–70 years	0.12	0.66	0.34	0.25	2.27	0.43	0.48	0.29	0.8	1.66	2.76	
2014 ^1^ [28]	Spain												3 × 24-h food recalls Own database [2,32]
	108 (54 w, 54 m)	0.08	0.41	0.23	0.25	3.04	0.31	0.32	0.14	0.74	2.29	5.29	
	Sample grouped												
	according age:												
	20–35 years (27 w, 27 m)	0.06	0.24	0.18	0.15	2.46	0.19	0.43	0.1	0.95	4.3	9.5	
	45–65 years (27 w, 27 m)	0.09	0.52	0.3	0.34	4.01	0.39	0.27	0.13	0.77	2.08	5.92	
	Sample grouped												
	according sex:												
	Women (27 w, 27 m)	0.08	0.47	0.24	0.24	3.39	0.35	0.33	0.14	0.69	2.36	4.93	
	Men (27 w, 27 m)	0.06	0.29	0.21	0.28	2.76	0.25	0.21	0.11	0.84	1.91	7.64	
2001 [26,27]	Spain												FFQ (108 items) European Carotenoid Database [26]
	64 (32 w, 32 m) 25–45 years	0.1	0.37	0.41	[0.54]	[5.51]	[2.46]	0.19	0.07	0.17	2.71	2.43	
	France												
	75 (38 w, 37 m) 25–45 years	0.15	0.71	0.22	1.38	10.88	0.81	0.11	0.07	0.28	1.57	4	
	Northern Ireland												
	65 (33 w, 32 m) 25–45 years	0.08	0.42	0.17	1.94	10.34	1.79	0.03	0.04	0.1	0.75	2.5	
	Republic of Ireland												
	73 (33 w, 40 m) 25–45 years	0.11	0.54	0.21	2.29	9.61	1.41	0.05	0.06	0.15	0.83	2.5	
	The Netherlands												
	72 (39 w, 33 m) 25–45 years	0.08	0.45	0.31	1.27	8.1	1.76	0.06	0.06	0.18	1	3	
	5 European countries (SP, FR, NI (UK), RI, NT)	0.09	0.49	0.25	1.57	8.89	1.65	0.06	0.06	0.152	1	2.53	
**Spanish subjects subsample** *n* = 348 mean ± SD, [median]		0.12 ± 0.04 [0.11]	0.53 ± 0.16 [0.54]	0.38 ± 0.12 [0.38]	0.39 ± 0.17 [0.39]	3.55 ± 1.39 [3.2]	1.02 ± 0.9 [0.65]	0.33 ± 0.12 [0.32]	0.18 ± 0.09 [0.17]	0.58 ± 0.28 [0.67]	2.07 ± 1.30 [1.98]	3.37 ± 1.30 [2.88]	
**Whole sample** *n* = 633 mean ± SD, [median]		0.12 ± 0.04 [0.11]	0.56 ± 0.13 [0.58]	0.34 ± 0.13 [0.30]	0.65 ± 0.63 [0.39]	4.4 ± 3.03 [3.21]	0.81 ± 0.61 [0.65]	0.3 ± 0.17 [0.32]	0.17 ± 0.10 [0.17]	0.57 ± 0.29 [0.67]	1.64 ± 0.53 [1.63]	3.96 ± 1.28 [4.72]	

^1^ Date of publication of the study design and results for the main outcome. Individual provitamin A carotenoids, intake and blood concentrations were calculated for the present study. w: women, m: male, α-car.: α-carotene, β-car: β-carotene, β-cx: β-cryptoxanthin, ^bio^: apparent bioavailability FCT: food composition table. SP: Spain, FR: France, NI (UK): Northern Ireland (United Kingdom), RI: Republic of Ireland, NT: Netherlands

**Table 2:** Dietary intakes and serum or plasma concentrations (means or [medians]) of α-carotene (α-car.), β-carotene (β-car.) and β-cryptoxanthin (β-cx.) from the literature (2011–2020).

		Mean Concentration in Blood (µmol/L) [Median]			Mean Dietary Intake (µmol/day) [Median]			Dietary Intake Assessment Method/FCT	Apparent Bioavailability			Ratio of Apparent Bioavailability	
Year [Ref.]	Country, n Subjects Sex, Age	α-Car.	β-Car.	β-Cx.	α-Car.	β-Car.	β-Cx.		α-Car.^bio^	β-Car.^bio^	β-Cx.^bio^	α-Car^bio^/β-car^bio^	β-Cx.^bio^/β-Car.^bio^
**EUROPE**													
2012 [36]	Europe, *n* = 856												
	(225 w, 631 m)		0.45			[4.3]		24-h recall		0.11			
	58.5 years							EPIC database					
2013 [37]	Sweeden, *n* = 159												
	Women, 56–75 years	0.08	0.46	0.51	1.92	6.46	0.83	FFQ (96 items) Various FCT	0.04	0.07	0.62	0.57	8.86
2016 [38]	UK, *n* = 30												
	Women, 18–30 years	-	0.6	0.18	2.72	9.76	0.58	FFQ	-	0.06	0.31	-	5.12
2017 [39]	UK												
	Men (*n* = 2362)	0.14	0.38	0.14	0.76	3.79	0.73		0.18	0.1	0.19	1.8	1.9
	Women (*n* = 2208)	0.19	0.5	0.2	0.75	3.79	0.82		0.25	0.13	0.24	1.92	1.85
								7-day food records					
	Men (*n* = 3817)	0.13	0.36	0.13	0.73	3.7	0.68		0.18	0.1	0.19	1.8	1.9
	Women (*n* = 3657)	0.18	0.48	0.19	0.73	3.65	0.77		0.25	0.13	0.25	1.92	1.92
2019 [40]	Spain, *n* = 156				0.37	1.78	0.17	(3×) 24-h record	0.38	0.25	1.47	1.52	5.88
	7–9 years	0.14	0.45	0.25									
					1.61	5.41	0.33	2 FFQ	0.09	0.08	0.76	1.13	9.5
								USDA and Spain Data (2008)					
**NORTH AMERICA**													
2011 [41]	USA, *n* = 250												
	W & M, 38 years												
	Afro-American	0.05	0.26	0.18	[0.62]	[2.85]	[0.19]	(4×) 24-h record	0.08	0.09	0.95	0.89	10.56
					[0.67]	[4.28]	[0.29]	NCI-DHQ	0.08	0.06	0.62	1.33	10.33
	White	0.08	0.33	0.16									
					[2.05]	[3.93]	[0.24]	(4×) 24-h record	0.04	0.08	0.67	0.5	8.38
					[1.05]	[6.00]	[0.28]	NCI-DHQ	0.08	0.06	0.57	1.33	9.5
2011 [42]	USA												
	Men, *n* = 1598	0.07	0.31	0.16		3.93				0.08			
	44–45 years							(2×) 24-h recall					
	Women, *n* = 1606 45–56 years	0.1	0.43	0.17		4.04				0.11			
2011 [16]	USA												
	*n* = 113	0.16	0.68	0.3	1.53	5.92	0.37	(3×) food record	0.11	0.12	0.81	1.14	9.25
	*n* = 116	0.11	0.5	0.25	1.15	5.72	0.23	FFQ	0.1	0.09	1.09	1.23	12.4
	W&M, 18–69 years												
2012 [43]	USA, *n* = 470												
	Women (217)	0.13	0.46	0.2	1.07	6.54	0.25	DHQ-24 h	0.12	0.07	0.8	1.76	11.37
	45–61 years				0.45	4.71	0.14	(2×) 24 h	0.3	0.1	1.42	3.04	14.5
	Men (253)	0.1	0.34	0.18									
	46–62 years				0.93	5.42	0.24	DHQ-24 h	0.11	0.06	0.73	1.76	11.64
					0.47	4.73	0.14	(2×) 24 h	0.22	0.07	1.26	3.04	17.46
								NDSR					
2012 [44]	USA, *n* = 6062												
	Men (3078) Women (2984)												
	2–19 years	0.04	0.2		0.4	2.01	0.26	24 h	0.1	0.1		1	
2013 [45]	USA, *n* = 1393 Women, 52–65 years												
		0.14	0.54	0.15	1.49	8.27	0.33	FFQ	0.09	0.07	0.46	1.29	6.57
2014 [46]	USA												
	*n* = 60				1.31	7.74	0.34	(30-day food records	0.15	0.04	0.71	3.75	17.75
	Women (40) Men (20)	0.19	0.32	0.24				(2×) FFQ					
	18–25 years				0.95	1.45	0.15	Nutrition Database System for Research	0.2	0.22	1.6	0.91	7.27
2016 [47]	USA and Canada												
	*n* = 909	0.17	0.67	0.25	1.04	6.33	0.25	24-h NBP	0.16	0.11	1	1.55	9.43
	Women and men 50–99 years				0.78	6.15	0.2	24-h BP	0.22	0.11	1.25	2	11.4
					2.05	11.47	0.33	FFQ NBP	0.08	0.06	0.76	1.43	13.07
					1.94	13.36	0.43	FFQ BP	0.09	0.05	0.58	1.76	11.62
2019 [48]	Canada, *n* = 265												
	Men (155), 44 years	[0.23]	[0.44]	[0.15]	4.73	18.81	1.74	Canadian Nutrient File	0.05	0.02	0.09	2.5	4.5
	Women (110), 41 years	[0.27]	[0.59]	[0.18]	2.66	11.81	1.36		0.1	0.05	0.13	2	2.6
2019 [49]	USA												
	NHANES 2003–2014 (diet), 2003–2006 (blood)	0.09	0.43	0.19	0.91	4.74	0.2	(2×) 24-h recalls	0.1	0.1	0.95	1	9.5
2019 [50]	USA, *n* = 136												
	Diabetics—type 1	0.19	0.63	0.38	0.7	3.14	0.24	(3×) 24-h record	0.27	0.2	1.58	1.35	7.9
	10.5–14.5 years							NDSR					
2019 [51]	USA												
	*n* = 80, 82 (diet, serum), women	[0.08]	[0.35]	[0.26]	[0.91]	[8.83]	[0.22]	FFQ	0.1	0.04	1.18	2.5	29.5
**ASIA**													
2016 [52]	Japan, *n* = 260												
	Men (173)		0.76		0.67	4.06	0.56	FFQ (115 items)		0.19			
	Women (87)		1.34		0.79	4.77	0.49			0.28			
	adults												
2018 [53]	China												
	*n* = 2947							FFQ (79 items) Chinese FCT 2004					
	Men (934)	0.06	0.4	0.12					0.03	0.03	0.5	1	16.7
	54–67 years				2.44	14.1	0.24						
	Women (2013)	0.08	0.59	0.18					0.03	0.04	0.75	1	18.75
	52–63 years												
**AUSTRALIA**													
2014 [54]	Australia												
	*n* = 150	0.07	0.35	0.14	3.37	15.73	1.07	FFQ	0.02	0.02	0.13	1	6.5
	(73 w, 77 m)							Nuttab 2006: Australian FCT					
	59–74 years												
2015 [55]	Australia												
	*n* = 38	0.12	0.76	0.14	[0.02]	[0.11]	[0.006]	FFQ (120 items) USDA-NCI	6	6.09	23.33	0.99	3.83
	(25 w, 13 m), 43 years												
**Mean ± SD [Median]** *		**0.13 ± 0.06** **[0.12]**	**0.5 ± 0.21** **[0.45]**	**0.21 ± 0.09** **[0.18]**	**1.32 ± 1.08** **[0.91]**	**6.12 ± 4.24** **[4.72]**	**0.76 ± 1.61** **[0.33]**		**0.37 ± 1.18** **[0.10]**	**0.32 ± 1.23** **[0.10]**	**1.21 ± 2.71** **[0.71]**	**1.61 ± 0.83** **[1.35]**	**9.2 ± 6.90** **[8.38]**

* These values have been calculated considering all the studies included in Table 2. α-car.: α-carotene, β-car: β-carotene, β-cx: β-cryptoxanthin, bio: apparent bioavailability, w: women, m: male, BP: black participants, DHQ: dietary-history food-frequency questionnaire, EPIC: European Prospective Investigation into Cancer and Nutrition FCT: food composition table, FFQ: food frequency questionnaire, NCI: National Cancer Institute, NDSR: Nutrition Data System for Research, NBP: non-black participants, USDA: United States Department of Agriculture, UK: United Kingdom.

**Table 3:** Major food contributors to provitamin A carotenoid intake.

		Major Food Contributors to Provitamin A Carotenoid Intake (% Contribution of Individual Items to the Total Intake of Each Carotenoid)		
Year [Ref.]	Subjects (n) Sex/Age	α-Carotene	β-Carotene	β-Cryptoxanthin
2020 [29]	Spain	Carrot (95)	Carrot (48)	Orange (32)
	(101: 77 w, 24 m)	Banana (3)	Tomato (12)	Orange juice: natural (23)
	45–65 years	Green beans (1,5)	Spinach (6)	concentrated (2)
			Tangerine (2)	Tangerine (2)
			Red pepper (2)	Red pepper (3)
2018 [30]	Spain	Carrot (95)	Carrot (48)	Orange (32)
	75 (w)	Banana (3)	Tomato (12)	Orange juice: natural (23)
	50–70 years	Green beans (1,5)	Spinach (6)	concentrated (2)
			Red pepper (1,5)	Tangerine (25)
				Red pepper (4)
2014 [28]	Spain			
	(108)	Carrot (88)	Carrot (34)	Orange (20)
	20–35 years (27w, 27m)	Banana (4)	Tomato (14)	Orange juice: natural (49), concentrated (6)
	45–65 years (27w, 27m)	Green beans (2)	Spinach (14)	Tangerine (16)
2001 [26,27]	Spain	Carrot (60)	Spinach (26)	Tangerine (53)
	(64: 32 w, 32m)	Tangerine (17)	Carrots (24)	Orange (38)
	25–45 years			
	France	Carrot (82)	Carrot (38)	Orange juice (50)
	(75: 38w, 37m)	Orange (6)	Spinach (14)	Orange (30)
	25-45 years			
	Northern Ireland	Carrot (88)	Carrot (53)	Orange juice (45)
	(65: 33 w, 32 m)	Coleslaw (6)	Soups (10)	Orange (26)
	25–45 years			
	Republic of Ireland	Carrot (90)	Carrot (60)	Orange (42)
	(73: 33w, 40 m)	Coleslaw (5)	Tomato products (13)	Tangerine (28)
	25–45 years			
	The Netherlands	Carrot (87)	Carrot (42)	Tangerine (41)
	(72: 39 w, 33 m)	Orange (5)	Spinach (12)	Orange juice (33)
	25–45 years			
2013 [45]	USA	Carrot	Carrot	Orange
	*n* = 1393 Women	Banana	Broccoli	Peach
	52–65 years		Lettuce	Apricot

w: women, m: male.
